# Gene expression and proteomic analysis of shoot apical meristem transition from dormancy to activation in *Cunninghamia lanceolata* (Lamb.) Hook

**DOI:** 10.1038/srep19938

**Published:** 2016-02-02

**Authors:** Huimin Xu, Dechang Cao, Yanmei Chen, Dongmei Wei, Yanwei Wang, Rebecca Ann Stevenson, Yingfang Zhu, Jinxing Lin

**Affiliations:** 1 Key Laboratory for Genetics and Breeding of Forest Trees and Ornamental Plants of Ministry of Education, College of Biological Sciences and Biotechnology, Beijing Forestry University, Beijing 100083, China; 2 College of Biological Sciences, China Agricultural University, Beijing 100193, China; 3 School of Life Science, Taizhou University, Zhejiang 318000, China; 4 Department of Horticulture and Landscape Architecture, Purdue University, West Lafayette, IN 47906, USA

## Abstract

In contrast to annual plants, in perennial plants, the shoot apical meristem (SAM) can undergo seasonal transitions between dormancy and activity; understanding this transition is crucial for understanding growth in perennial plants. However, little is known about the molecular mechanisms of SAM development in trees. Here, light and transmission electron microscopy revealed that evident changes in starch granules, lipid bodies, and cell walls thickness of the SAM in *C. lanceolata* during the transition from dormancy to activation. HPLC-ESI-MS/MS analysis showed that levels of indole-3-acetic acid (IAA) increased and levels of abscisic acid (ABA) decreased from dormant to active stage. Examination of 20 genes and 132 differentially expressed proteins revealed that the expression of genes and proteins potentially involved in cell division and expansion significantly increased in the active stage, whereas those related to the abscisic acid insensitive 3(ABI3), the cytoskeleton and energy metabolism decreased in the dormant stage. These findings provide new insights into the complex mechanism of gene and protein expression and their relation to cytological and physiological changes of SAM in this coniferous species.

Stem cells play a critical role in the regulation of growth and development of multicellular species. In plants, the shoot apical meristem (SAM) comprises a small population of stem cells, self-renewing cells characterized by asymmetric cell division. The progeny of these stem cells differentiate into diverse specialized cell types, which can form different organs and tissues, such as stems, leaves, branches, and flowers^1^,^2^,^3^. In many tree species, the SAM forms part of the apical bud, which positioned at the tips of shoots, producing cells for tip growth. The progeny of the SAM stem cells form the entire above-ground structure of plant^4^; similarly, the progeny of the root apical meristem form the below-ground structure of the plant.

In contrast to annual plants, the SAM in perennial plants can continue to grow for many years. In plants growing in temperate and boreal regions, meristems can transition between dormant and active states, altering plant growth in response to environmental conditions; this transition involves morphological, physiological and genetic regulatory changes^5^, with dormant cells generally showing reduced metabolic activity and tissue development^6^,^7^. The regulation of dormancy has primary significance for productivity and survival in perennial plants^8^, and it responds to intrinsic (phytohormone and molecular mechanisms) and extrinsic signals (environmental control), such as temperature and so on^8^,^9^,^10^. In trees, release from dormancy, sometimes described as “bud burst”, involves visible event indicating the end of winter dormancy and growth start, which largely affect the annual production and is a complex processes^11^.

Over the past decades, our understanding of the regulation of meristem dormancy has expanded rapidly. Accumulating evidence shows that the SAM exhibits seasonal changes in morphology, water, and starch content; for example, bud water contents increased with release from dormancy and decreased in dormancy^12^. Moreover, the carbohydrate metabolism was demonstrated in crown buds of leafy spurge (*Euphorbia esula*) during the seasonal shifts of dormancy status, and the mechanisms underlying processes such as cell wall biochemistry, or responsive to auxin were also differentially regulated^13^. Endogenous hormonal changes in cytokinins, auxins and abscisic acid (ABA) concentration were involved in the bud dormancy induction and release process^14^,^15^,^16^. Recently, extensive transcriptome sequencing and proteome approaches have examined the molecular events of dormancy in apical buds in *Populus tremula × Populus alba*^17^, *Picea glauca*^18^, *Pyrus pyrifolia*^19^. Although considerable progress has been made in revealing the physiological and molecular aspects of bud dormancy, few studies have examined gene and protein expression and their relationship to cytological and physiological changes during the transition from dormant to active stage.

*Cunninghamia lanceolata* Lamb. Hook. is the most important and fast-growing coniferous evergreen tree in terms of both industrial and commercial wood supplies in China^20^. To understand the growth and development of this key tree species, we have intensively examined its stem cells^21^,^22^,^23^. On the basis of accumulation of our intensive investigations over the years, this study is part of a series examining the plant stem cell of the coniferous tree. Here, to elucidate key changes in gene expression and protein abundance, we examined gene expression levels and the proteome during the dormancy-activity transition in SAM of *C. lanceolata*. In parallel, we also examined cytological and physiological changes, thereby providing new insights into the molecular mechanisms that regulate SAM development in this coniferous species.

## Results

### Cytological and physiological changes in the apical buds

**Figure 1:**
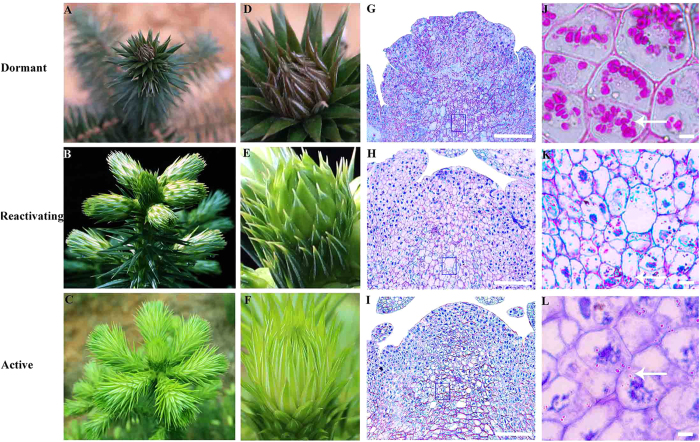
Changes in morphology and polysaccharides in apical buds of *C. lanceolata* during the dormancy-activity transition. Overview of the shoot apical meristem (SAM) in the field (**A–C**); bud morphology at dormant, reactivating, and active stages (**D–F**); distribution of polysaccharides in buds at different stages (**G–I**); enlarged view of G, H, I (**J–L**). Scale bars: 100 μm in G–L. The white arrows indicate the polysaccharides.

For this study, we divided the apical buds into dormant, reactivating, and active development stages, based on their morphology (Fig. 1A–C). In dormancy, the buds are yellowish-brown and tightly enclosed by waxy sheaths (Fig. 1D); the reactivating buds are tender, green, and not tightly enclosed, but have partially separated to sprout and reveal primordial shoots (Fig. 1E). In active buds, the scales have fallen off and the bud shows evident, continuous growth (Fig. 1F).

**Figure 2:**
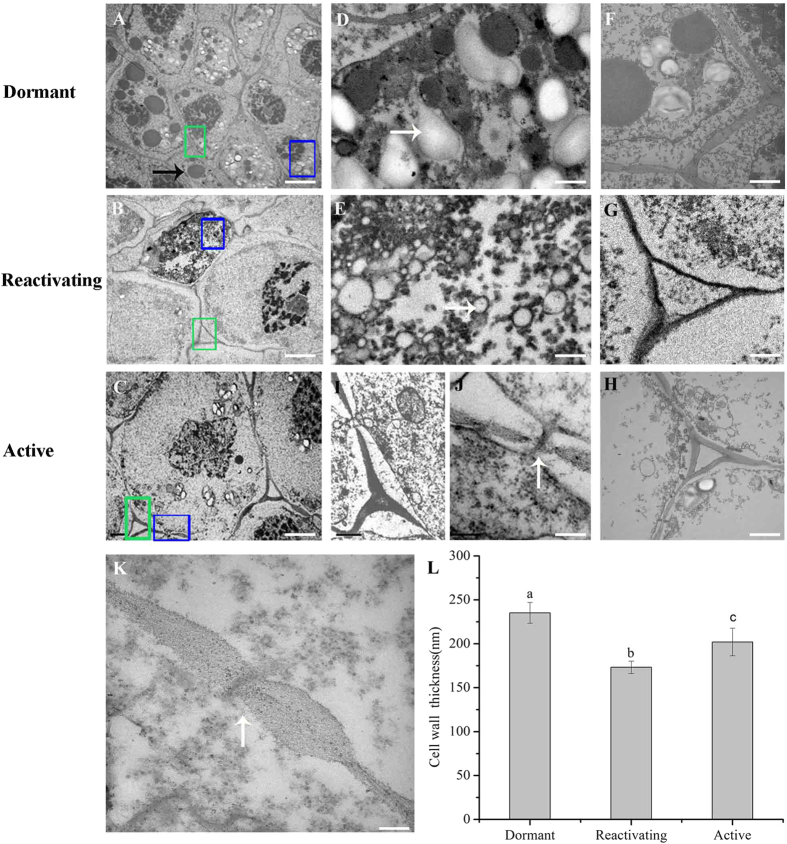
Transmission electron micrographs of *C. lanceolata* buds during the dormancy-activity transition. The distribution of starch granules and lipid bodies at the dormant, reactivating, and active stages (**A–C**). The dormant buds had large lipid bodies (**A,D**) and thicker cell walls (**F**) compared with buds at the reactivating (**G**) and active stages (**H**). (**I**) and (**J**) show visible plasmodesmata (PD) in active stage buds. The cell corner also shows more compact at dormant stage (**F**); (**I**) and (**J**) show visible plasmodesmata (PD) in active stage buds. The plasmodesmata structure between dormant and active stage was obviously different (**J,K**). Plasmodesmata sphincters were not present between cell and cell at active stage (**J**). The thickness of cell wall in dormant, reactivating and active stages showed significant difference (**L**). Bars: 2 μm in A to C, 500 nm in (**D**) to (**E**), 1 μm in (**G**) to (**L**) and 100 nm in (**I,J**). The white and black horizontal arrows represent the starch granules and the lipid bodies, respectively. Vertical arrows indicate the plasmodesmata (PD). The blue boxes show the amplified region of Fig. 2D, Fig. 2E and Fig. 2I, respectively. The green boxes zoom in the region of Fig. 2F, Fig. 2G and Fig. 2H.

To examine the bud growth and physiological state, we first used Periodic Acid Schiff (PAS) staining to examine the polysaccharide contents of the cells. We observed that a large number of starch granules in the dormant buds, displaying the polysaccharides characteristics (Fig. 1G,J), and fewer starch granules in reactivating buds (Fig. 1H,K), but increased numbers of granules in active buds (Fig. 1I,L), which was confirmed by transmission electron microscopy (Fig. 2A–C). In addition, abundant of lipid bodies were visible at the dormant stage (Fig. 2A, D), but only rarely were present in the reactivating and active buds (Fig. 2B,C). We further found that the lipid bodies were abundant in dormant buds, while also decreased with significantly at reactivating stage than those at dormant stage (Fig. 2D,E). Furthermore, we observed that the cell wall was much thicker at dormant stage compared to that in reactivating and active stage (Fig. 2F–H). The thickness of cell wall was about 1.35-fold (P < 0.05) at the dormant stage compared with that at active stage (Fig. 2L). The plasmodesmata structure between dormant and active stages was obviously different (Fig. 2I,J,K). Plasmodesmata sphincters were not present between cell and cell at active stage (Fig. 2J).

**Figure 3:**
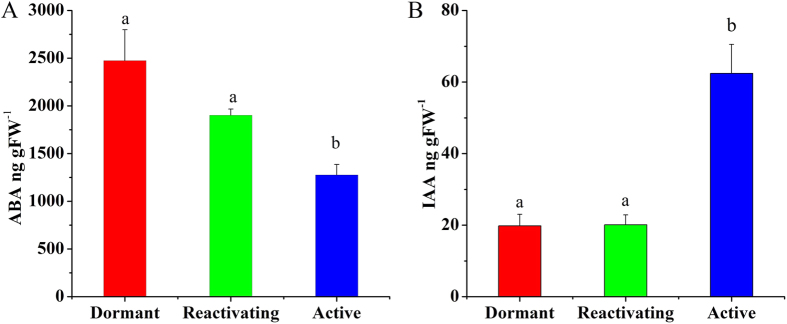
The changes of abscisic acid (ABA) and indole acetic acid (IAA) in apical bud of *C.lanceolata.* Data are means with 3 technical replicates each, with error bars representing standard deviation (SD). Non-overlapping letters (a–c) indicate significant difference (p < 0.05) from each other. Error bars represent standard errors.

Changes in plant hormone levels often occur with alterations in bud activity^12^. In parallel with the evident cytological changes of SAM development, therefore, we analyzed that the changes of hormone during the dormancy-activity transition in *C.lanceolata* by HPLC-ESI-MS/MS analysis. The ABA concentration significantly decreased, by about two-fold (P < 0.05), from the dormant to the active stage (Fig. 3A). By contrast, the IAA concentration increased by about three-fold (P < 0.05) from the dormant to the active stage (Fig. 3B).

### Gene expression changes associated with cell proliferation

**Figure 4:**
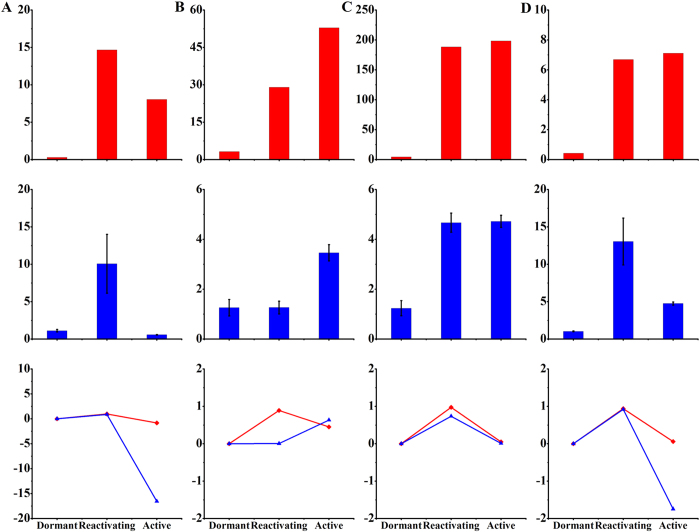
Expression of cell proliferation related genes in SAM and vascular cambium. Values of expression levels (RPKM) by RNA-seq were standardized in vascular cambium (red bars). Relative expression of bud-dormancy related genes was determined by quantitative real-time RT-PCR (blue bars). The data represent mean value ± SD from three biological replicates. Asterisk indicates significant differences (P ≤ 0.05). The Y-axes show expression levels and the (**A–D**) from left to right on the X-axis represent genes encoding histone 4 (**A**), class III homeodomain leucine zipper protein (**B**), PIN-like auxin efflux carrier (**C**) and auxin-induced protein 5NG4 **(D**). The line chart indicated the variation degree of these genes between in SAM (blue lines) and vascular cambium (red lines) during the dormancy-activity transition.

**Figure 5:**
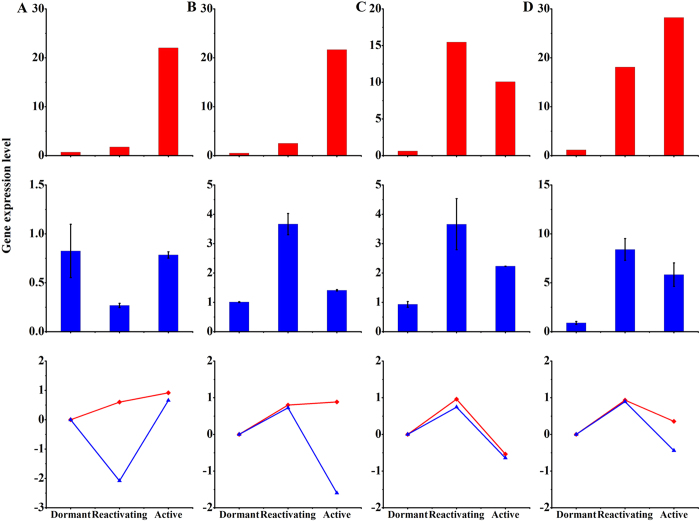
Expression of cell division related genes in SAM and vascular cambium. Values of expression levels (RPKM) from RNA-seq were standardized in vascular cambium (red bars). Relative expression of bud-dormancy related genes was determined by quantitative real-time RT-PCR (blue bars). The data represent mean value ± SD from three biological replicates. Asterisk indicates significant differences (P ≤ 0.05). The Y-axes show expression levels and the (**A–D**) from left to right on the X-axis represent genes encoding zinc finger protein (**A**), R2R3-MYB transcription factor MYB2 (**B**), A-like cyclin (**C**), and ARF-L1 protein (**D**).The line chart indicated the variation degree of these genes between in SAM (blue lines) and vascular cambium (red lines) during the dormancy-activity transition.

**Figure 6:**
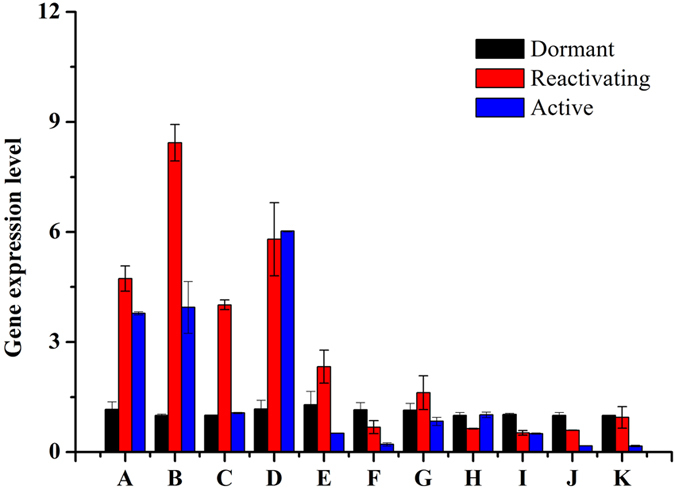
Genes expression in apical buds during the dormancy-activity transition. Relative expression of bud-dormancy related genes was determined by quantitative real-time RT-PCR. The data represent mean value ± SD from three biological replicates. Asterisk indicates significant differences (P ≤ 0.05). A to K of the X axis represent putative genes beta-1,3-glucanase, xyloglucan endotransglycosylase, endo-beta-1,4-glucanase, pectate lyase, ABI3-interacting protein 2, sugar transporter family protein, peroxidase-like protein, AP2-EREBP, WRKY, Pm WRKY115, Pm WRKY 117.

Based on our previous investigation of gene expression in the vascular cambium of *C. lanceolata*^22^, we selected highly conserved genes involved in cell division and these genes show similar developmental changes in the apical meristem and vascular cambium during the transition from dormant to active stage, which has been reported in previous reports^1^. In our investigation, we found that the genes expression associated with cell proliferation changes significantly in apical buds during the dormancy-activity transition. Moreover, we particularly compared the changes of these genes between in apical buds and vascular cambium (Fig. 4). We further found that reactivating buds showed the highest expression of the genes encoding histone 4 (Fig. 4A), PIN-like auxin efflux carrier (Fig. 4C) and auxin-induced protein 5NG4 (Fig. 4D), which are involved in cell division and cell expansion in the SAM and vascular cambium. By contrast, active buds showed the highest expression levels of the gene encoding the class III homeodomain leucine zipper protein (Fig. 4B) and. In addition, the levels of the transcript encoding zinc finger protein of SAM declined from the dormant to the reactivating stage, and then increased from the reactivating stage to the active stage (Fig. 5A). Some genes associated with cell proliferation, such as R2R3-MYB transcription factor MYB2 (Fig. 5B), A-like cyclin (Fig. 5C) and ARF-L1 protein (Fig. 5D) showed the highest expression level in the reactivating stage and decreased significantly in dormant buds, consistent with the slower rate of cell division and expansion during dormancy. Strikingly, expression level for the negative regulatory factor ABI3-interacting protein 2, significantly declined from the reactivating to the active stage (Fig. 6).

### Gene expression changes associated with cell wall modification and energy

The cell wall undergoes dynamic modification under different growth conditions^24^,^25^,^26^, therefore, we hypothesized that the seasonal changes of the SAM that give rise to distinct morphological characteristics might also affect the cell wall. To test this hypothesis, we analyzed genes associated with cell wall structure and cell wall expansion by qRT-PCR. The results showed that the expression of genes encoding beta-1,3-glucanase, xyloglucan endotransglycosylase hydrolase, endo-beta-1,4-glucanase, and pectate lyase obviously increased from the dormant stage to the reactivating stage, and then slightly decreased from the reactivating stage to the active stage in apical bud of *C.lanceolata*. (Fig. 6). We further found that the expression of sugar transporter family genes in the dormant bud was higher than in the reactivating and active buds (Fig. 6). Similarly, the expression of the gene encoding peroxidase-like protein increased from the dormant to the reactivating stage, but decreased from reactivating stage to active stage. By contrast, the genes expression of AP2-EREBP showed a slight increase from the dormant stage to active stage. We also found that genes related to the cold stress, such as WRKY, PmWRKY115 and PmWRKY117 declined during the dormancy-activity transition (Fig. 6).

### Differential protein expression in apical buds

**Figure 7:**
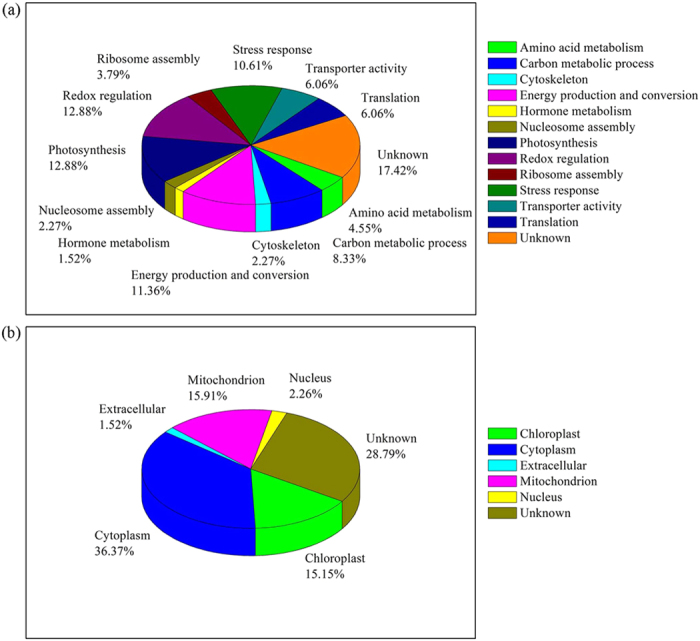
Functional classification and distribution (**a**); Protein subcellular locations (**b**).

To elucidate the underlying developmental mechanisms of apical buds, we used a label-free proteomic approach to systemically analyze the dynamic changes in the bud proteome. The observed peptide intensities were optimized and normalized to an internal standard by using the Protein Lynx Global Server (PLGS) auto normalization function. To determine protein expression, we performed three biological replicates of LC-MS measurements for each stages. We identified 391 proteins, among which, 132 showed significant changes at the three developmental stages (Table S1 in Additional file 1). The differentially expressed proteins can be classified into 13 functional groups according to their molecular function, biological process, and cellular component based on annotations in AgBase (www.agbase.msstate.edu/) and UniProtKB (www.uniprot.org/uniprot/)(Fig. 7 and Table S1 in Additional file 1). These proteins were mainly involved in photosynthesis, redox regulation, energy production and conversion, stress response, and cytoskeleton (Fig. 7A). According to the subcellular localization analysis, these 132 proteins were located in the cytoplasm (36.8 %), chloroplast (15.0 %), mitochondrion (15.8 %), extracellular (1.5%), nucleus (2.3%) and 28.6% of the proteins lacked exact localization annotations (Fig. 7B).

**Figure 8:**
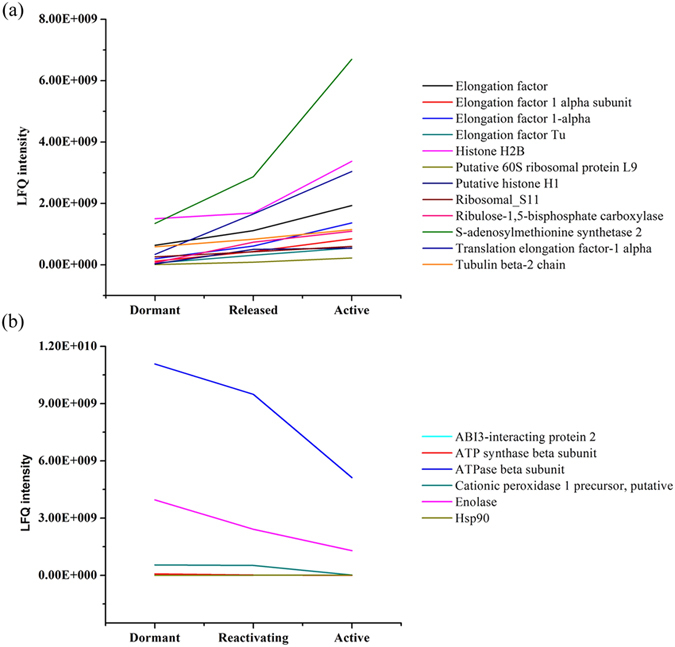
Differential protein expression of SAM in *C.lanceolata* during dormancy-activity transition. The up-regulated (**a**) and down-regulated (**b**) proteins. The up-regulated proteins including the elongation factor (AAK85129.1), elongation factor 1 alpha subunit (CAA11705.1), elongation factor 1-alpha (XP_002312029.1), elongation factor Tu (AAS58429.1), histone H2B (ABK26056.1), putative 60S ribosomal protein L9 (BAD07825.1), putative histone H1(BAE92289.1), ribosomal_S11 (ABB87125.1), ribulose-1,5-bisphosphate carboxylase (AAN71862.1), S-adenosylmethionine synthetase 2 (Q9FVG7.1), translation elongation factor-1 alpha (CAC27139.1) and tubulin beta-2 chain (EEC74344.1) (**a**); the down-regulated proteins including ABI3-interacting protein 2 (AAP31312.2), ATP synthase beta subunit (CAF21946.1), ATPase beta subunit (AAD03393.1), cationic peroxidase 1 precursor (XP_002517727.1), Glutathione peroxidase (ABQ96600.1), enolase (Q43130.1) (**b**).

We compared the levels of the 132 differentially expressed proteins regulating cell proliferation and differentiation in apical buds and found that several of these proteins were up-regulated to higher levels in active stages. For example, we observed higher levels of the elongation factor (AAK85129.1), elongation factor 1 alpha subunit (CAA11705.1), elongation factor 1-alpha (XP_002312029.1), elongation factor Tu (AAS58429.1), histone H2B (ABK26056.1), putative 60S ribosomal protein L9 (BAD07825.1), putative histone H1(BAE92289.1), ribosomal_S11 (ABB87125.1), ribulose-1,5-bisphosphate carboxylase (AAN71862.1), S-adenosylmethionine synthetase 2 (Q9FVG7.1), translation elongation factor-1 alpha (CAC27139.1) and tubulin beta-2 chain (EEC74344.1) (Fig. 8A). Comparing the reactivating and active stages with the dormant stage, we also observed higher levels of proteins involved in translation, such as 60S ribosomal protein L9 (BAD07825.1) and the elongation factors (Fig. 8A). On the other hand, ABI3-interacting protein 2 (AAP31312.2), ATP synthase beta subunit (CAF21946.1), ATPase beta subunit (AAD03393.1), cationic peroxidase 1 precursor (XP_002517727.1), and Glutathione peroxidase (ABQ96600.1) belonging to energy production and conversion were all down-regulated dramatically and decreased from dormant stage to reactivating stage (Fig. 8B). In addition, the cytoskeleton group including enolase (Q43130.1) and profilin (O81982.1) were also down-regulated dramatically (Fig. 8B and Table S1 in Additional file 1).

## Discussion

### Cytological and physiological changes in the transition between dormancy and activity

The dormancy-activity transition is part of adaptation to seasonal growth in perennial plants and we observed significant morphological and physiological changes in buds undergoing this transition. In apical bud development of Norway spruce, the high sugar contents in dormant buds decreased with the release of dormancy^27^. It was found that numerous lipid bodies emerged in the shoot apex of perennial plants during seasonal growth arrest, and appeared to function in dormancy release by reconstituting cell-cell signaling paths in the apex^28^,^29^. Our results showed that the maximum accumulation of starch and lipid bodies occurred in the bud dormancy, along with the characteristic yellowish-brown and tightly enclosed bud morphology, suggesting that the accumulation of polysaccharides and lipids may improve the adjustments to response to the seasonal change.

Plant hormones are known to be important in plant growth and plant development^14^,^30^. Previous studies showed that IAA in buds of ‘Anna’ apple trees may affect transcription of nuclear DNA^31^ and IAA increased during bud growth in *Phaseolus vulgaris* L. cv Tender Green^16^. Furthermore, high concentrations of ABA coincided with bud dormancy, and the decreased level of ABA in active stage may contribute to the rapid bud burst and vigorous growth^32^. In the present experiment, we uncovered that there was an increase level of IAA from dormant to active stage and the IAA level reached its maximum in the active stage. Furthermore, we found a decrease in ABA levels from the dormant to the active stage, with the highest ABA in dormant apical buds, intermediate levels in reactivating buds, and lowest levels in active buds, providing strong evidence that IAA and ABA function in distinct stages of bud dormancy.

### Genes and proteins associated with cell proliferation

Cell division and cell elongation predominantly govern plant growth^33^. Some histone, such as H2A.Z, variants have been reported function in transcriptional control of bud dormancy^34^. In *A. thaliana*, H2A.Z variant (AtHTA8; ABD5) are more highly expressed in buds close to bud burst^35^. In *Rubus idaeus*, histone transcripts also are abundant at dormancy release^36^ and Histone 2A is up-regulated at bud break in *Vitis riparia*^37^. The present investigation revealed that the histone H2B (ABK26056.1) increased by 1.34-fold during the transition from dormant to active stage, in agreement with the function of histones in cell division and growth. Moreover, we found that histone H4, A-like cyclin and ARF-L1 protein were significantly increased from the dormant to reactivating stage.

In hybrid aspen, the dormancy-activity transition in the cambial meristem involves stage-specific modulation of the auxin response^38^. In the present investigation, in addition to the analysis of the ABA and IAA contents, we also analyzed the genes encoding PIN-like auxin efflux carrier and auxin-induced protein 5NG4, showing higher expression at the reactivating stage than the dormant stage. Importantly, by qRT-PCR analysis, we found that the homologs of genes involved in auxin response in apical buds were highly similar to those in the vascular cambium during the transition from dormant to reactivating stage reported in our previous study in Chinese fir^22^. Given that similar trends in the abundance of histones, transcription factors, and auxin-related proteins occur in the apical meristem and the vascular cambium of Chinese fir, our findings suggest that some genes related to cell division have conserved functions in apical and lateral meristems.

The abundance of S-adenosylmethionine synthase (AdoMetS) positively correlated with cell division activity^39^. In the apical buds of *Pinus sylvestris* L. var. *mongolica litv*., the higher expression of S-adenosylmethionine synthetase during April, indicates its involvement in the initiation of bud dormancy release^40^. In this experiment, we found an increase of S-adenosylmethionine from dormant stage to active stage, and higher expression (2.27-fold change) in active than in dormant buds, indicating that S-adenosylmethionine affects cell proliferation. Numerous studies have also showed that the induction of protein synthesis and activity could be closely related to bud dormancy^41^,^42^. In *Pinus sylvestris* L. var. *mongolica litv*, elongation factor 1 (EF-1) is a component of the protein synthesis machinery and a prerequisite for maintaining rapid cell division and protein synthesis in tissues such as meristematic tissues or somatic embryos^43^. Interestingly, the eukaryotic initiation factor and the translation elongation factor EF-1b2 were down-regulated by ABA, and the expression of the elongation factor Tu was up-regulated during dormancy release^44^. As a transcription factor in the ABA signal transduction pathway, ABI3 appears to function in seed dormancy^45^. Moreover, it has been reported that ABI3 influences bud development possibly by modifying ABA sensitivity^17^. Our findings revealed that elongation factor Tu (AAS58429.1) increased 3.26-fold during the transition from dormancy to active growth. However, the ABA concentration decreased about two-fold, similar to the changes of the gene encoding negative regulatory factor ABI3-interacting protein 2. Furthermore, the expression changes of ABI3-interacting protein 2 (AAP31312.2) also showed similar tendency, implying its connection with hormonal and gene expression changes. Based on these results, it is reasonable for us to speculate that elongation factor Tu affects the translation of mRNAs required for cell division in the shoot meristem, and can be used as a marker of the meristematic state of a cell.

### Genes and proteins associated with cell wall remodeling

The different elastic properties of the cell wall in the shoot apical meristem are linked to growth rates^46^. Cell-wall enlargement begins with wall stress relaxation, causing the cells to take up water and enlarge. Most genes associated with cell wall expansion, such as xyloglucan endotransglycosylases, expansins and endo-(1,4)- beta-glucanases, are up-regulated in the active region of the meristem^47^. In oak (*Quercus robur* L.), the abundant expression of the xyloglucan endotransglycosylase (*XTH*) gene observed in the endodormancy library also suggested a potential role in cold acclimation, through the modification of cell wall structure^5^. *XTH* modifies xyloglucan, the main hemicellulose found in primary cell walls of pine trees^47^,^48^. In the present study, *XTH* expression was up-regulated during the transition from dormancy to release, and the expression of genes encoding beta-1,3-glucanase, endo-1,4-beta-glucanase and pectate lyase showed large increases from dormancy to release in Chinese fir. Cytologically, we found that the cell wall in the dormant bud was much thicker than that in the reactivating bud and the cell corner was more compact in the dormant stage. Based on these findings, we can conclude that these genes related to cell wall remodeling participate in bud response to seasonal changes, bridging data collected at the morphological level to the molecular changes.

### Genes and proteins associated with the cytoskeleton

Morphogenesis and the cytoskeleton have a close functional relationship. Beta-tubulin, the basic structural unit of microtubules required for cell division and cell elongation, is considered a marker for monitoring dormancy in trees^49^,^50^. Indeed, the accumulation of beta-tubulin associated with breaking of seed dormancy and germination was shown in *Acer platanoides*^51^. The present results showed that tubulin beta-2 (EEC74344.1) was upregulated by 1.3-fold in the transition from dormancy to activity. Moreover, the dormant buds showed strong accumulation of profilin, which act in changes of the cytoskeleton; this observation has not been reported in other experiments. Based on these results, we conclude that genes related to the cytoskeleton are involved in the response of bud development to seasonal changes, and are strongly linked to the morphological and molecular changes causing bud dormancy.

### Genes and proteins associated with energy metabolism

In general, dormancy is considered to be a state of reduced metabolic activity when many cellular processes remain inactive^8^. The transition from bud dormancy to active growth involves numerous molecular and biochemical changes, including extensive reconfiguration of carbohydrate metabolism^52^. Increasing evidence demonstrated the SAM requires sufficient energy from the underlying tissue to sustain bud growth in the time of dormancy release^40^,^53^,^54^. Recently, several proteomic and transcriptomic studies have shown that energy metabolism is essential for leaf and flower development, including dormancy release. In the present investigation, the 132 differentially expressed proteins were found to be associated with dormancy release, the functional classes mainly involving photosynthesis, redox regulation, energy production and conversion, stress response and cytoskeleton, accounting for 12.6%, 12.6%, 11.1%, 10.4% and 8.1% of the differentially expressed proteins. Combined with previous researches, our results elucidated the importance of respiration and the biosynthesis of metabolic compounds in plants, especially woody plants. In hybrid poplar, energy production and conversion was required to protect buds from frost damage during dormancy transition^7^. The accumulation of Rubisco in apical buds could also induce the plant to begin carbon fixation during the dormancy release^18^. Enolase, a multifunctional enzyme related to glycolysis, can act as a transcription factor especially involved in cold tolerance^53^. Based on our label-free quantitative protein analysis, we found that enolase (Q43130.1) and the ATP synthase beta subunit (CAF21946.1) had higher expression at the dormant stage than other stages. In addition, the ribulose-1, 5-bisphosphate carboxylase (AAN71862.1) was upregulated by 3.94-fold from bud dormancy to early release. Importantly, we validated by qRT-PCR that transcripts for sugar transporter family proteins were also more abundant in dormant buds than buds in reactivating and active stage of Chinese fir, confirming that the requirement for adequate energy in dormancy and bud break.

The adaptation and acclimation of plants to repeated episodes of abiotic and biotic stresses was even more crucial. *In Arabidopsis*, there was a direct link between glutathione biosynthesis and stress defense gene expression, reflecting the physiological state of the plant once an environmental challenge^55^. Many WRKY genes act in plant defense responses^56^. Since we demonstrated that the AP2-EREBP, WRKY, PmWRKY115 and PmWRKY117 all changed noticeably and showed significantly higher expression level at dormant stage than at active stage, it is reasonable to speculate that these genes are required for the reconfiguration of energy metabolism to improve the freezing tolerance of buds and enable them to survive winter.

## Conclusions

Here, we examined changes in gene expression and protein abundance in apical bud development, in parallel with morphological and physiological alterations, during the dormancy-activity transition in Chinese fir. We find that: (1) the changes in ABA and IAA indicated they function in distinct stages of growth during the dormancy-activity transition; (2) some highly conserved genes involved in cell division show similar developmental changes in the apical meristem and vascular cambium, implying that they exert a similar role in the regulation of both types of stem cells; and (3) the reconfiguration of energy metabolism likely affects the apical bud development underlying the dormancy-activity transition. Taken together, the data presented here substantially increase our understanding of the complex regulatory framework of apical meristem development in woody plants.

## Materials and Methods

### Plant materials

Apical buds at dormant, reactivating, and active stages were collected from 3-year-old *C. lanceolata* growing under natural conditions in Minhou County (N26°35′, E119°24′), Fujian Province, China. Samples were collected on 25 January 2013, 25 March 2013 and 27 May 2013, to cover the major development stages of the bud dormancy, dormancy reactivating and activity. Apical buds were harvested, frozen in liquid nitrogen, and were then stored at −80 °C.

### Light microscopy and Transmission electron microscopy (TEM)

Apical buds were fixed with 2.5% (v/v) glutaraldehyde in 100 mM sodium phosphate buffer (pH 7.2) and post fixed with 1% osmium teroxide. After successive ethanol dehydration, and infiltration the shoot-tips were embedded into Spurr’s resin. Sectioning was performed using a Leica microtome. Sections (2 μm) were mounted on slides, stained with 0.25% (w/v) toluidine blue O (Sigma), and observed under a Zeiss Axioskop 2 Plus microscope equipped with a computer-assisted digital camera. Images were processed using Photoshop (Adobe, San Jose, CA, USA). Ultra-thin sections (70 nm thick) were cut on an ultramicrotome (Leica Ultracut R, Leica, Germany), stained with uranyl acetate (1%), and observed under a transmission electron microscope (JEM-1230, JEOL, Japan). The polysaccharide was stained by Periodic Acid Schiff (PAS) reaction for 30 min at room temperature. Three replicate sections were investigated from each bud sample. The cell wall thickness was also measured in the three stages using NIH Image-J.1 software (http://rsb.info.nih.gov).

### Hormone analysis by HPLC-ESI-MS/MS

Apical buds were analyzed for endogenous levels of indole-3-acetic acid and abscisic acid. Apical buds were ground into powder (50 mg) and were transferred to 2ml tubes, with 500 μl extraction solvent (2 propanol: 1 H_2_O: 0.002 concentrated HCl), and then various volumes of internal standards solution were added. After that the tubes were shaken at 100 rpm for 30 min at 4 °C, and then 1 ml dichloromethane was added to each sample, which was then shaken for 30 min at 4 °C and centrifuged at 13,000 g for 5 min at 4 °C. After centrifugation, the lower phase was transferred into a screw-cap vial and concentrated using a nitrogen evaporator. The samples were redissolved in 100 μl methanol, and 1 μl of sample solution was injected into the reverse-phase C18 Gemini HPLC column for HPLC–ESI–MS/MS analysis^57^.

Data are means with 3 technical replicates each, with error bars representing standard deviation. Non-overlapping letters (a–c) indicate significant difference between different stage buds, based on ANOVA analysis and Multiple Range Tests procedure with a confidence level of 95%.

### Quantitative real time-polymerase chain reaction (qRT-PCR)

Total RNAs from dormant, reactivating, and active buds were isolated using Concert Plant RNA Reagent (Invitrogen) following the supplied protocol. First-strand cDNA synthesis was performed from 1 μg of total RNA and oligo (dT) primers with Superscript II reverse transcriptase (Invitrogen) according to the manufacturer’s instructions. PCR amplifications were performed in 20 μl volumes, containing SYBR Green master mix. All reactions were performed in triplicates and were analyzed on an ABI 7500 Sequence Detection System (Applied Biosystems) with a first step of 95 °C for 2 min followed by 45 cycles of 95 °C for 15 s and 60 °C for 1 min. Melting curves were generated using the following program: 95 °C for 15 s, 60 °C for 15 s, and 95 °C for 15 s. Absolute values for gene expression were obtained using a Fluor-S Imager and integrated quantization software (ABI). Transcript abundance was quantified using standard curves for both target and reference genes, which were generated from serial dilutions of PCR products from corresponding cDNA. The results were expressed relative to the expression levels of an internal reference gene, glyceraldehyde 3-phosphate dehydrogenase (*GAPDH*, CV170251), in each sample using the 2^−ΔΔ*C*t^ method^58^.

### Protein extraction and in-solution digestion

Proteins were extracted from Chinese fir buds as previously described with some modifications^7^. Samples were ground in liquid nitrogen and dissolved in extraction buffer (500 mM Tris-HCl, 25% sucrose (w/v), 2% ß-mercaptoethanol, 50 mM EDTA, 1 mM DTT, 2 mM PMSF, 0.1 M KCl, pH 8.0) per g of tissue fresh weight in a 3 ml falcon tube, and shaken for 10 min at 4 °C. Equal volumes of chilled water saturated phenol were added and the incubation was shaken for 15 min at 4 °C. After centrifugation at 12,000 rpm and 4 °C for 15min, the soluble proteins were dissolved in the upper phenolic phase (the high sucrose concentration causing a phase inversion). The phenolic phase was separated and the proteins precipitated overnight in four volumes of ice-cold acetone. After centrifugation at 12000 rpm and 4 °C for 20 min the pellets were washed three times with ice-cold methanol. The dried protein pellets were dissolved in a buffer containing 8 M urea, 100 mM NH_4_HCO_3_ by gentle shaking at room temperature for 5 min. The concentration of protein solutions was determined by 2-D Quant Kit (GE Healthcare) with bovine serum albumin (BSA) as a solution. Protein digestion was modified from previous described^59^. The pH of the samples was adjusted to 8.5 using 1 M ammonium bicarbonate, and about 50 μg protein was used for chemical reduction of each sample. The trypsin digest was incubated at 37 °C for 14 h. The peptide mixture was acidified using 1 μl formic acid for further MS analysis.

### Protein identification by LC-MS and data analysis

For protein identification, at least three fragment ions were observed per peptide with at least two peptides identified per protein. The maximum false positive rate allowed was 4%. Label-free quantitation was performed in MaxQuant. The quantitative changes in protein levels were analyzed using Waters Expression ^E^, and the parameters for the quantitative analysis have been explained in detail previously^60^. The quantification of protein was analyzed with data sets normalized with the PLGS “auto-normalization” function, where the data are normalized to the intensity of the many qualitatively matched proteins (or peptides) whose abundances do not change between conditions as established by statistical analysis. Included limits were all protein hits that were identified with a confidence of >95%. Identical peptides from each triplicate set per sample were clustered based on mass precision (typically ~5 ppm) and a retention time tolerance of <0.25 min using clustering software included in PLGS 2.3. Only those proteins identified in at least two of three injections and having fold changes >1.3 were regarded as having undergone a significant change^60^.

## Additional Information

**How to cite this article**: Xu, H. *et al*. Gene expression and proteomic analysis of shoot apical meristem transition from dormancy to activation in *Cunninghamia lanceolata* (Lamb.) Hook. *Sci. Rep.* **6**, 19938; doi: 10.1038/srep19938 (2016).

## Supplementary Material

Supplementary Information
