# Supplementary material for: Gene expression and proteomic analysis of shoot apical meristem transition from dormancy to activation in Cunninghamia lanceolata (Lamb.) Hook
Source: Sci Rep. 2016 Feb 2;6:19938. doi: 10.1038/srep19938 (PMC4735791; doi:10.1038/srep19938)
Supplement: Supplementary Information [file srep19938-s1.pdf]

Gene expression and proteomic analysis of shoot apical meristem transition from dormancy to activation in *Cunninghamia lanceolata* (Lamb.) Hook.

Huimin Xu[1], Dechang Cao[1], Yanmei Chen[2], Dongmei Wei[3], Yanwei Wang[1], Rebecca Ann Stevenson[4], Yingfang Zhu[4], Jinxing Lin[1*]

Table S1 Differentially expressed proteins identified by LC-MS^E

Accession no.	Protein name	Log R/D bud	P value	Log A/ D bud	P value	Log A/ R bud	P value
**Amino acid metabolism**							
**ABK24266.1**	Putative uncharacterized protein	inf	0.31	#N/A	#N/A	-1.87	0.29
**ABR17712.1**	Putative uncharacterized protein	inf	0.18	inf	0.21	-1.75	0.10
**ABK22122.1**	Putative uncharacterized protein	1.03	0.00	1.31	0.19	0.28	0.63
**ACN39946.1**	Putative uncharacterized protein	-0.77	0.56	0.84	0.21	1.61	0.17
**CAA53078.1**	3-ketoacyl-coa thiolase B	0.73	0.65	-1.30	0.41	-2.03	0.40
**Q9FVG7.1**	S-adenosylmethionine synthetase 2	2.31	0.01	2.27	0.00	-0.04	0.88
**Carbon metabolic process**							
**AAM81204.1**	Fructose-1,6-diphosphate aldolase	1.74	0.09	1.77	0.02	0.03	0.95
**ACU23111.1**	Fructose-bisphosphate aldolase	-1.60	0.13	0.17	0.58	1.77	0.10
**ACU21446.1**	Fumarylacetoacetase	1.06	0.06	-0.54	0.68	-1.60	0.15
**CAC80374.1**	Glyceraldehyde-3-phosphate dehydrogenase	1.87	0.17	2.27	0.00	0.40	0.49
**AAA34077.1**	Glyceraldehyde-3-phosphate dehydrogenase	2.63	0.10	1.48	0.43	-1.15	0.30
**ABP68613.1**	Malic enzyme	-3.34	0.09	-inf	0.13	#N/A	#N/A
**AAP68889.1**	Putative glyoxysomal malate dehydrogenase	inf	0.24	#N/A	#N/A	-1.48	0.41
**BAF46028.1**	Putative Adomet decarboxylase	2.09	0.01	1.98	0.00	-0.12	0.68
**XP_002520198.1**	Pyruvate dehydrogenase, putative	-1.30	0.11	-1.49	0.06	-0.19	0.87
**XP_002520198.1**	Pyruvate dehydrogenase, putative	-1.30	0.11	-1.49	0.06	-0.19	0.87
**AAT80327.1**	UDP-D-glucuronate decarboxylase	3.58	0.01	3.27	0.04	-0.31	0.52
**Cytoskeleton**							
**O81982.1**	Profilin	-2.14	0.33	-inf	0.37	#N/A	#N/A
**EEC74344.1**	Tubulin beta-2 chain	0.96	0.11	1.31	0.12	0.34	0.57
**XP_002283198.1**	Putative uncharacterized protein	1.57	0.00	1.64	0.05	0.07	0.86
**Energy production and conversion**							
**XP_001771011.1**	Phosphoribulokinase	1.79	0.27	2.18	0.03	0.39	0.64
**BAD45421.1**	Putative cytosolic phosphoglycerate kinase 1	0.98	0.07	1.30	0.00	0.32	0.29
**XP_002321126.1**	Aconitate hydratase	inf	0.00	#N/A	#N/A	-2.37	0.01
**Q43130.1**	Enolase	-1.61	0.04	-1.62	0.04	0.00	0.99
**CAF21946.1**	ATP synthase beta subunit	-3.17	0.27	-inf	0.39	#N/A	#N/A
**BAH73398.1**	ATP synthase CF1 alpha subunit	1.26	0.35	1.60	0.34	0.35	0.78

Accession no.	Protein name	Log R/D bud	P value	Log A/ D bud	P value	Log A/ R bud	P value
**P31541.1**	ATP-dependent Clp protease ATP-binding subunit clpa homolog CD4A, chloroplastic	2.55	0.19	3.03	0.04	0.49	0.55
**AAC06385.1**	Early fruit mrna	inf	0.15	inf	0.17	-2.33	0.21
**CAJ42303.1**	VDAC-like porin	-1.42	0.31	-1.46	0.30	-0.04	0.97
**AAD03393.1**	ATPase beta subunit	-1.30	0.01	-1.11	0.01	0.18	0.48
**ABR16190.1**	Cyt_c_oxidase_su6a	-1.48	0.29	-1.11	0.40	0.37	0.76
**AAP80659.1**	Dynamin like Pr6(ADL6)	inf	0.19	#N/A	#N/A	-1.62	0.33
**AAR27291.1**	Peptidyl-prolyl cis-trans isomerase	-1.47	0.01	-1.03	0.03	0.44	0.32
**XP_002320625.1**	Succinyl-coa ligase beta-chain family protein	-3.09	0.01	-2.44	0.03	#N/A	#N/A
**XP_001765853.1**	Predicted protein	0.14	0.90	-1.29	0.40	-1.43	0.22
**Hormone metabolism**							
**AAP31311.2**	ABI3-interacting protein 1	-inf	0.01	-1.54	0.14	#N/A	#N/A
**AAP31312.2**	ABI3-interacting protein 2	-1.69	0.34	-1.92	0.29	#N/A	#N/A
**Nucleosome assembly**							
**ABK24519.1**	Putative uncharacterized protein	-inf	0.47	-3.43	0.43	#N/A	#N/A
**ABK26056.1**	Histone H2B	1.17	0.10	1.34	0.14	0.18	0.72
**BAE92289.1**	Putative histone H1	4.27	0.40	#N/A	#N/A	-inf	0.38
**Photosynthesis**							
**AAB41553.1**	6-phosphogluconate dehydrogenase, decarboxylating	inf	0.18	#N/A	#N/A	-3.30	0.05
**A4QK31.1**	Cytochrome f	3.37	0.10	2.68	0.21	-0.68	0.51
**BAH73335.1**	Cytochrome f	1.29	0.11	1.93	0.03	0.64	0.18
**CAB60127.1**	Cytosolic phosphoglucomutase	2.97	0.22	1.94	0.05	-1.03	0.47
**XP_002273783.1**	Lyceraldehyde-3-phosphate dehydrogenase B, chloroplastic isoform 2	1.41	0.29	1.80	0.01	0.39	0.56
**ACM51422.1**	Mg-protoporphyrin IX chelatase	2.21	0.03	1.70	0.32	-0.51	0.48
**XP_002307234.1**	O2 evolving complex 33kd family protein	-0.87	0.12	-1.91	0.06	-1.04	0.22
**NP_042469.1**	Photosystem II 44 kda protein	3.24	0.30	1.58	0.40	-1.67	0.37
**BAH73339.1**	Photosystem II CP47 chlorophyll apoprotein	4.10	0.26	2.86	0.08	-1.24	0.40
**AAX76828.1**	Photosystem II subunit A	inf	0.30	inf	0.20	-1.36	0.46
**ACA30289.1**	Putative ribulose-1,5-bisphosphate carboxylase/oxygenase activase alpha 2	5.34	0.04	5.16	0.38	-0.18	0.89
**AAA63163.1**	Ribulose 1,5-bisphosphate carboxylase activase isoform 1	3.28	0.00	2.78	0.41	-0.50	0.65

Accession no.	Protein name	Log R/D bud	P value	Log A/ D bud	P value	Log A/ R bud	P value
**AAG22772.1**	Ribulose-1,5-biphosphate carboxylase/oxygenase large subunit	-0.08	0.96	#N/A	#N/A	-3.09	0.11
**AAN71862.1**	Ribulose-1,5-bisphosphate carboxylase	3.94	0.22	3.49	0.02	-0.45	0.65
**AAW29427.1**	Ribulose-1,5-bisphosphate carboxylase/oxygenase large subunit	inf	0.15	#N/A	#N/A	-2.47	0.16
**AAL11902.1**	Ribulose-1,5-bisphosphate carboxylase/oxygenase large subunit	3.11	0.28	1.62	0.33	-1.48	0.42
**AAS88137.1**	Ribulose-1,5-bisphosphate carboxylase/oxygenase large subunit	1.86	0.07	1.65	0.17	-0.21	0.76
**Redox regulation**							
**ABK23157.1**	Putative uncharacterized protein	2.68	0.05	1.32	0.61	-1.36	0.27
**XP_002283310.1**	Mitochondrial-processing peptidase subunit alpha	-2.81	0.03	-1.93	0.09	#N/A	#N/A
**CAB71293.1**	Chloroplast ferredoxin-NADP+ oxidoreductase	1.26	0.10	1.35	0.01	0.09	0.81
**AAQ14591.1**	Copper/zinc superoxide dismutase	-2.42	0.20	-2.18	0.21	0.24	0.73
**XP_002528162.1**	Glycine cleavage system h protein, putative	-inf	0.14	-2.52	0.15	#N/A	#N/A
**CAN63539.1**	Hypothetical protein VITISV_016625	2.47	0.07	1.73	0.14	-0.75	0.26
**AAA96137.1**	Peroxidase	#N/A	#N/A	0.16	0.94	1.47	0.58
**ACD56608.1**	Putative protein disulfide	-1.78	0.36	-1.93	0.34	-0.16	0.89
**ACN39940.1**	Putative uncharacterized protein	-1.60	0.32	-1.12	0.41	0.49	0.10
**ABK24614.1**	Putative uncharacterized protein	-1.40	0.01	-1.22	0.01	0.18	0.66
**ABR16663.1**	Putative uncharacterized protein	2.28	0.01	2.13	0.04	-0.16	0.70
**ABR16034.1**	Putative uncharacterized protein	-1.50	0.56	#N/A	#N/A	-inf	0.18
**ABK21331.1**	Putative uncharacterized protein	-1.35	0.03	-1.52	0.04	-0.17	0.69
**ABR16661.1**	Putative uncharacterized protein	#N/A	#N/A	1.35	0.40	inf	0.30
**XP_001785162.1**	Putative uncharacterized protein	0.73	0.28	-0.56	0.62	-1.29	0.06
**EEE62118.1**	Glutathione_s-trfase_n	-4.60	0.44	#N/A	#N/A	-inf	0.30
**AAK27264.1**	Isoflavone reductase-like protein CJP-6	-1.43	0.13	-1.81	0.11	-0.38	0.07
**Ribosome assembly**							
**ACN40331.1**	Cyanate hydratase	-1.52	0.37	-2.17	0.29	-0.65	0.52
**XP_002510412.1**	Protein yrdA, putative	-2.05	0.01	-3.05	0.01	-1.00	0.53
**ABB87125.1**	Ribosomal_S11	1.21	0.17	1.49	0.08	0.28	0.47
**ABR17947.1**	Putative uncharacterized protein	2.48	0.03	2.65	0.01	0.18	0.59
**EEH59263.1**	Predicted protein	inf	0.27	#N/A	#N/A	-3.21	0.25
**Stress response**							
**AAR12193.1**	Heat shock protein 90	2.21	0.23	0.27	0.90	-1.93	0.26
**XP_002517727.1**	Cationic peroxidase 1 precursor, putative	-4.18	0.35	-5.26	0.33	-1.08	0.49

Accession no.	Protein name	Log R/D bud	P value	Log A/ D bud	P value	Log A/ R bud	P value
**ACT82479.1**	Chloroplast stromal ascorbate peroxidase 12	-1.42	0.13	-1.85	0.06	-0.44	0.57
**XP_001760558.1**	Cysteine_dsase	-2.01	0.09	-3.61	0.02	-1.60	0.12
**ACN60402.1**	Flavanone 3-beta-hydroxylase	#N/A	#N/A	1.79	0.45	2.34	0.38
**ABQ96600.1**	Glutathione peroxidase	-0.89	0.12	-2.39	0.03	-1.50	0.21
**ABJ80949.1**	Hsp90	inf	0.10	inf	0.26	-2.01	0.11
**Q9STX5.1**	Endoplasmin homolog	1.50	0.07	1.57	0.15	0.07	0.90
**XP_002447413.1**	Hypothetical protein SORBIDRAFT_06g000660	2.10	0.06	2.13	0.12	0.03	0.96
**BAA93024.1**	Putative phosphogluconate dehydrogenase	inf	0.18	inf	0.20	-2.04	0.22
**ABK25734.1**	Putative uncharacterized protein	inf	0.18	#N/A	#N/A	1.99	0.54
**ABK24283.1**	Putative uncharacterized protein	2.10	0.04	2.68	0.13	0.59	0.51
**ACN39800.1**	Unknown	2.41	0.00	2.25	0.06	-0.15	0.72
**ABK26682.1**	Putative uncharacterized protein	0.56	0.40	1.62	0.16	1.06	0.29
**Translation**							
**AAK85129.1**	Elongation factor	1.62	0.01	1.60	0.24	-0.02	0.98
**CAA11705.1**	Elongation factor 1 alpha subunit	2.84	0.02	2.88	0.21	0.04	0.96
**XP_002312029.1**	Elongation factor 1-alpha	2.45	0.07	2.77	0.22	0.32	0.72
**BAD26337.1**	Elongation factor 2	2.31	0.08	1.37	0.01	-0.94	0.13
**AAS58429.1**	Elongation factor Tu	3.26	0.02	2.77	0.01	-0.49	0.30
**BAD07825.1**	Putative 60S ribosomal protein L9	4.54	0.00	4.52	0.00	-0.02	0.95
**BAE92290.1**	Putative elongation factor	-2.00	0.28	-2.41	0.24	-0.41	0.75
**CAC27139.1**	Translation elongation factor-1 alpha	3.16	0.01	3.08	0.17	-0.07	0.93
**Transporter activity**							
**NP_001065986.1**	Non-specific lipid-transfer protein 2B	0.64	0.43	1.82	0.34	1.18	0.44
**1109190A**	Calmodulin	-2.61	0.37	-2.55	0.38	0.06	0.91
**P02662.2**	Antioxidant peptide	-inf	0.06	-1.59	0.18	#N/A	#N/A
**XP_002528311.1**	Clathrin heavy chain, putative	inf	0.21	#N/A	#N/A	-1.74	0.33
**BAF08021.2**	Os02g0184200	3.31	0.34	#N/A	#N/A	-2.36	0.34
**XP_002456080.1**	Putative uncharacterized protein Sb03g030060	-0.81	0.27	-1.50	0.17	-0.69	0.67
**XP_002310404.1**	Uncharacterized protein	inf	0.27	#N/A	#N/A	-1.49	0.44
**ABR17902.1**	Putative uncharacterized protein	-2.83	0.03	-2.55	0.03	0.29	0.26
**Unknown**							
**XP_002276912.1**	Hypothetical protein isoform 2	2.09	0.04	2.98	0.15	0.89	0.43

Accession no.	Protein name	Log R/D bud	P value	Log A/ D bud	P value	Log A/ R bud	P value
**XP_002531232.1**	Transcription factor, putative	-inf	0.19	-1.78	0.29	#N/A	#N/A
**ACN40912.1**	Uncharacterized protein	inf	0.01	#N/A	#N/A	-2.33	0.02
**ACN40697.1**	Unknown	-1.40	0.12	-1.56	0.07	-0.16	0.83
**ACU20577.1**	Unknown	-1.85	0.15	-1.65	0.11	0.20	0.78
**ACN40685.1**	Unknown	-1.79	0.20	-2.52	0.20	-0.73	0.45
**ACN40755.1**	Unknown	3.98	0.21	#N/A	#N/A	-inf	0.30
**ACN41066.1**	Unknown	-2.82	0.01	-inf	0.04	#N/A	#N/A
**ACJ85785.1**	Unknown	2.63	0.00	2.88	0.05	0.25	0.64
**ACN40584.1**	Unknown	2.19	0.17	2.40	0.28	0.21	0.84
**ACU23974.1**	Unknown	1.10	0.42	1.50	0.29	0.40	0.72
**ABR18217.1**	Putative uncharacterized protein	1.28	0.38	1.47	0.47	0.19	0.90
**ABK26810.1**	Unknown	1.48	0.07	1.45	0.24	-0.03	0.96
**ACN40726.1**	Unknown	-2.15	0.16	-1.49	0.21	0.66	0.67
**ACN40616.1**	Unknown	-0.96	0.02	-1.51	0.05	-0.55	0.45
**CAO65998.1**	Unnamed protein product	2.56	0.01	2.13	0.21	-0.42	0.56
**CAV26532.1**	Unnamed protein product	1.60	0.02	1.48	0.26	-0.12	0.87
**CAY34137.1**	Unnamed protein product	-2.89	0.31	-2.08	0.37	0.81	0.73
**CAO43976.1**	Unnamed protein product	-2.05	0.42	-2.19	0.42	-0.13	0.86
**CAW84864.1**	Unnamed protein product	-5.04	0.01	-3.92	0.00	1.12	0.39
**CAY38961.1**	Unnamed protein product	3.30	0.00	2.76	0.40	-0.54	0.62
**CAV26040.1**	Unnamed protein product	3.58	0.04	2.69	0.22	-0.89	0.34
**CAV25021.1**	Unnamed protein product	-2.87	0.42	-3.80	0.39	-0.93	0.19

Note: R, reactivating bud; D, dormant bud; A, active bud. The fold-change of different stages of bud described by log scale (1.3-fold-change). The positive and negative value represents that the fold-change of up-regulated and down-regulated differential proteins, respectively.
